# Supplementary material for: Diamond Blackfan anemia is mediated by hyperactive Nemo-like kinase
Source: Nat Commun. 2020 Jul 3;11:3344. doi: 10.1038/s41467-020-17100-z (PMC7334220; doi:10.1038/s41467-020-17100-z)
Supplement: Supplementary file 1 — Supplementary Information [file 41467_2020_17100_MOESM1_ESM.pdf]

Supplementary Figures

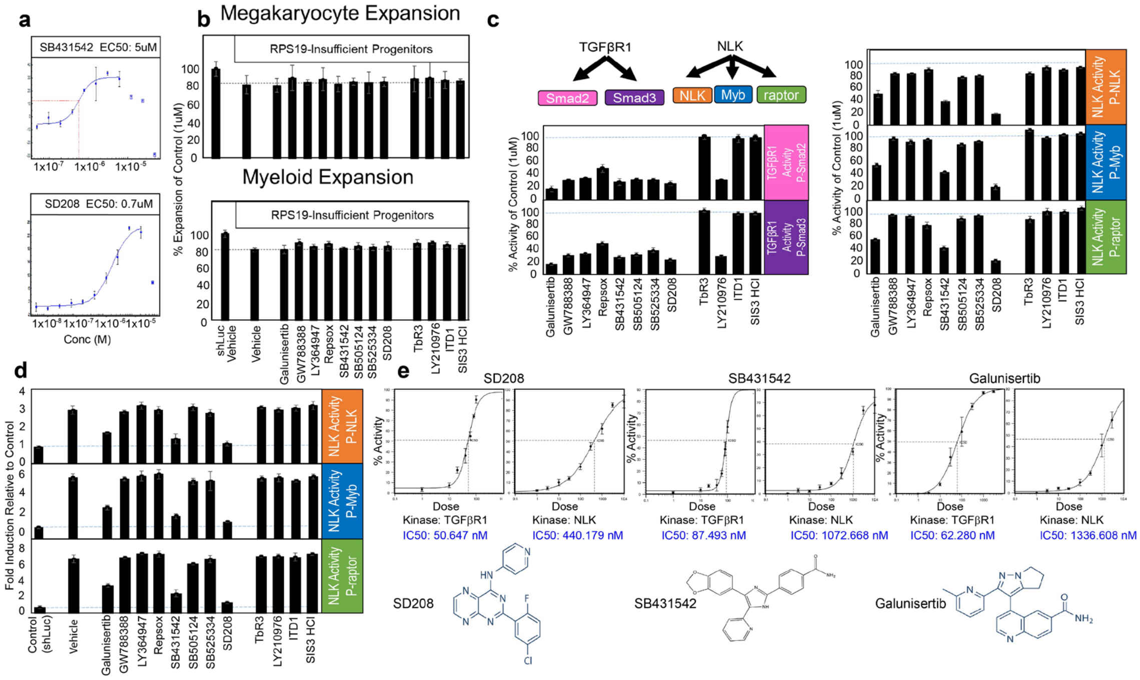

Supplementary Fig. 1. SB431542 and SD208 mediate dose-dependent rescue of RPS19-deficient erythroid progenitor expansion and have off-target inhibition of NLK. (a) SD208 and SB431542 were tested for their ability to rescue the proliferation defect of fetal liver erythorid cells from shRPS19 mice at a range of concentrations. Dose response curves for both compounds are shown. X-axis shows concentration of the compound dissolved in DMSO (Molar). (b) Differentiating cord blood CD34+ progenitors were transduced with shRNA against RPS19 and treated with inhibitors at working concentrations for TGFβ inhibition every 3 days. Cells were counted and CD41a+ megakaryocyte (upper) and CD11b+ myeloid cells (lower) were assessed by flow cytometry. Data was normalized to progenitors that were transduced with shRNA against luciferase (control). (c) Schematic indicating Smad2 and Smad3 serve as substrates for activated TGFβR1 while NLK, c-Myb and raptor serve as substrates for activated NLK. (Left panels) K562 cells were stimulated with 5 ng/ml TGFβ1 for 10 min and TGFβR1 was immunopurified after cell lysis and protein normalization. TGFβR1 was added to purified Smad2 (upper panel) and Smad3 (lower panel) in the presence of ATP and Mg^{2+} at 37°C for 30 min. Phosphorylation of substrates was detected by antibody raised against phosphorylated serine residues. Compounds with inhibitory properties against TGFβR1 or other TGFβ pathway factors were added to the kinase reactions at concentrations known to inhibit TGFβ signaling. The most potent stimulator of murine erythropoiesis (SD208) is depicted with a star. (Right panels) Active NLK was immunopurified from Kp53A1 cells cultured at 32°C for 24h and added to NLK substrates NLK (upper), c-Myb (middle) and raptor (lower), in the presence of the same inhibitory compounds. (d) Differentiating cord blood $CD34^{+}$ progenitors were transduced with shRNA against luciferase or RPS19 and treated with inhibitors at working concentrations for TGFβ inhibition. After 5 days NLK was immunopurified and NLK kinase activity assessed. (e) IC_{50} curves and values were calculated for SD208, SB431542 and Galunisertib against TGFβR1 and NLK *in vitro*. Structures of the compounds are included.

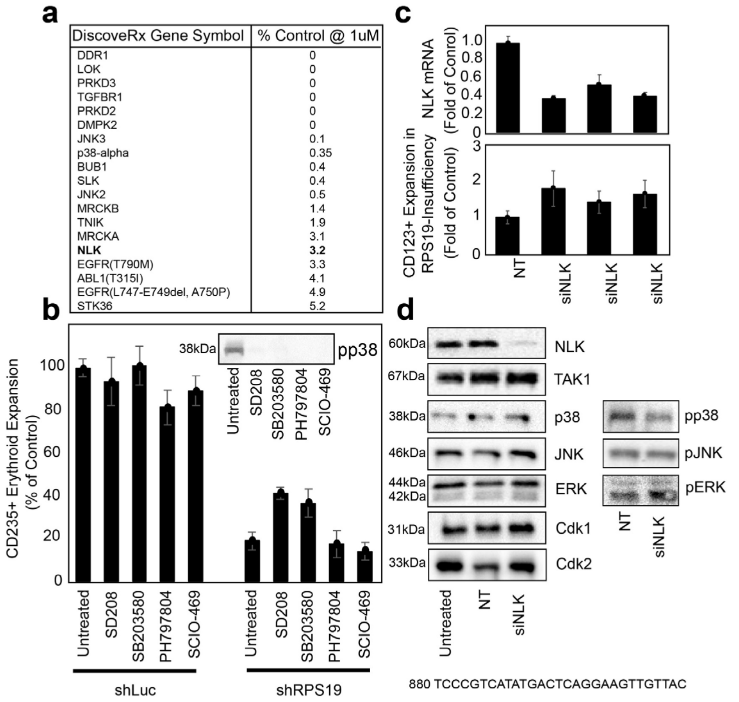

Supplementary Fig. 2. SD208 and NLK siRNA are specific. (a) The top 22 hits from DiscoveRX kinase profile analysis of SD208. (b) Human cord blood CD34+ progenitors were transduced shRNA against luciferase (shLuc) or RPS19 (shRPS19) and differentiated in erythroid media in the presence or absence of recommended concentrations of SD208, SB203580, PH797804 and SCIO-469 for 12 days. Cells were counted and assessed for cell surface expression of CD235. Effective inhibition of p38 activity was assessed by lysis of treated controls and Western blot analysis of pp38. (c) Cord blood CD34+ progenitors were transduced with shRNA against RPS19 and either non-targeting (NT) or 1 of 3 different shRNA sequences against NLK. After sorting, cells were differentiated for 12 days. Lysates were subjected to qRT-PCR to assess NLK expression (Upper) and the number of CD235+ erythroblasts was determined by flow cytometry and is expressed relative to erythroid expansion of control cells (Lower). (d) Kp53A1 cells were untransfected (Unt), transfected with a non-targeting sequence (NT), or siRNA against NLK (siNLK). Phosphorylation status of p38, JNK and ERK was determined by treating transfected Kp53A1 cells EPO prior to Western blot analysis for pp38, pJNK and pERK. Transfected cells were cultured for 48 hours prior to lysis. Lysates were subjected to SDS-PAGE and protein expression of the listed proteins was determined by Western blot analysis. The sequence of the siRNA is listed below the Western blot panels.

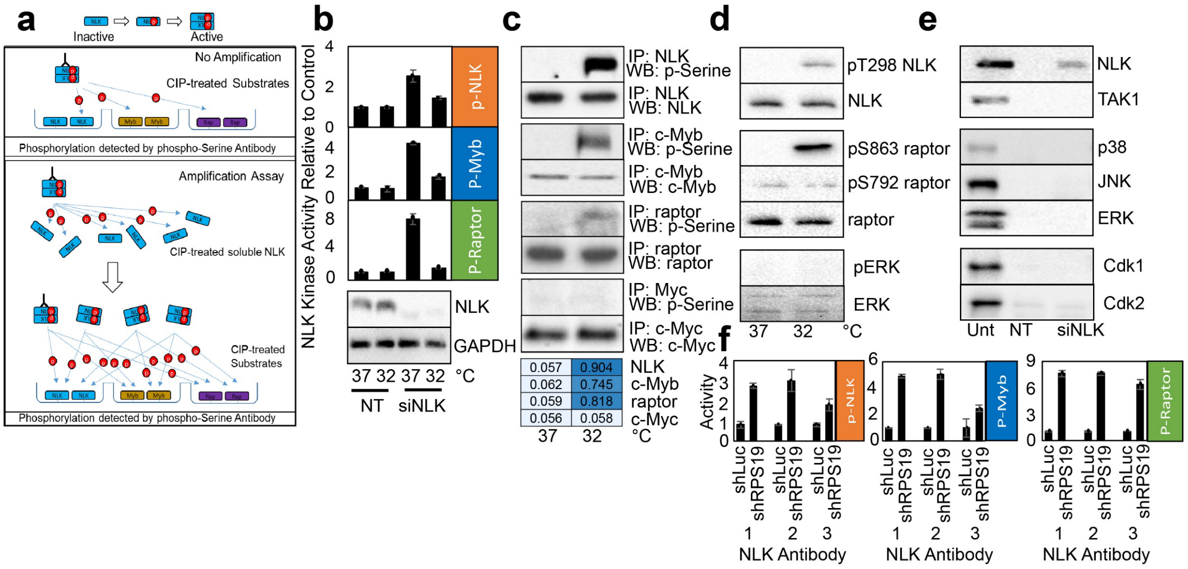

Supplementary Fig. 3. *In vitro* kinase assay phosphorylation is specific. (a) Schematic depicting NLK kinase assay. The upper diagram indicates the initial version that is limited to a minimum of approximately 1x10^5 cells for accurate values, while the lower diagram includes an additional amplification step that improves sensitivity down to 1x10^3 cells. Calf intestine phosphatase (CIP) was used to dephosphorylate immunopurified proteins that were biotinylated and bound to streptavidin-coated plates. As active NLK can dimerize and activate inactive NLK, if less than 1x10^5 cells were lysed, immunopurified NLK was incubated with purified, dephosphorylated NLK to increase the number of active NLK molecules above the threshold of detection (NB: the percentage of active NLK remains constant). (b) Kp53A1 cells stably expressing siRNA against NLK (siNLK) or a non-targeting control (NT) were grown at 37 or 32°C for 24h and immunoprecipitated NLK was subjected to kinase assay examining phosphorylation of NLK, c-Myb and raptor. Concurrently, cells were lysed and probed for NLK and GAPDH expression by Western blot. (c upper) NLK, c-Myb, Raptor and c-Myc were immunopurified from Kp53A1 cells grown at 37 or 32 °C for 24 h. After SDS-PAGE separation, samples were probed by Western blot with the same antibodies, or an antibody recognizing phosphorylated serine and threonine residues. (c lower) Readout of plate reader after in vitro NLK kinase assay examining absorbance at 428nm. (d) Kp53A1 cells were treated as above, except lysates were probed with the indicated phosphosite-specific and total antibodies. (e) Kp53A1 lysates were loaded directly (12ug) or 300ug was immunoprecipitated with IgG or anti-NLK antibody overnight at 4°C before being subjected to SDS-PAGE and Western blot analysis for the indicated proteins. (f) CD34+ HSPCs were differentiated as above and pooled into 3 separate aliquots. NLK was immunopurified using 3 distinct NLK antibodies recognizing unique epitopes and subjected to the *in vitro* NLK kinase assay.

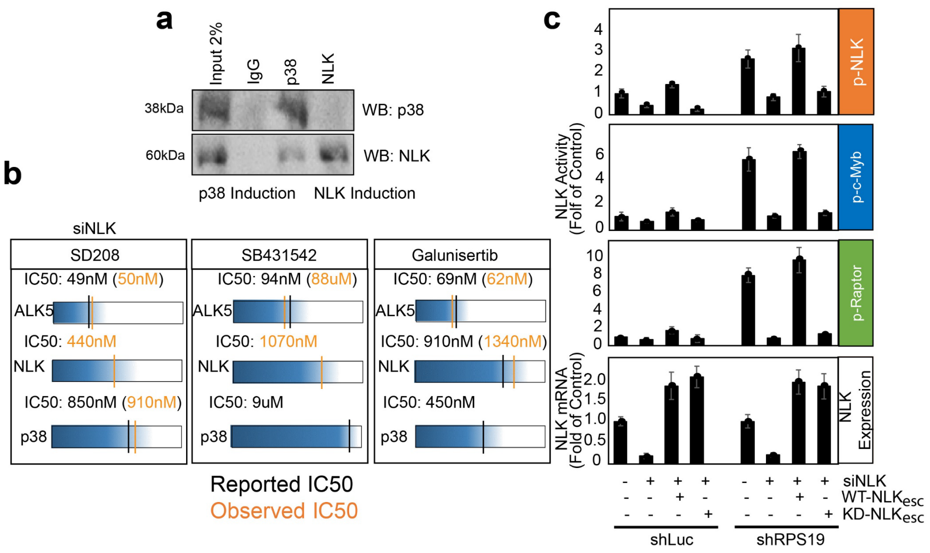

Supplementary Fig. 4. NLK mediated phosphorylation is not due to NLK associated p38 or other associated kinase. (a) Kp53A1 were lysed using the same lysis conditions utilized in our *in vitro* kinase assay. 500 µg of protein was subjected to immunoprecipitation at 4°C overnight with IgG, or antibodies raised against p38 or NLK. Immunoprecipitates were subjected to SDS-PAGE and probed by Western blot analysis for p38 or NLK. As a positive control, 2% of the lysate (input) was included. (b) IC_{50}s were calculated for SD208, SB431542 and Galunisertib against activated TGFβR1, NLK and p38. Observed values are in orange while reported are in blue. (c) NLK was immunoprecipitated from control (shLuc) and RPS19-insufficient (shRPS19) differentiating progenitors derived from CD34+ cord blood HSPCs at day 7. In addition of shRNA against RPS19 or control, cultures were transduced with siRNA against NLK alone, or with either a wild type (WTesc) or kinase-deficient (KDesc) siRNA-resistant NLK cDNA (with intact 3'UTR). NLK activity was assessed by *in vitro* kinase assay examining NLK, c-Myb and Raptor as substrates. Simultaneously, NLK expression was assessed by qRT-PCR.

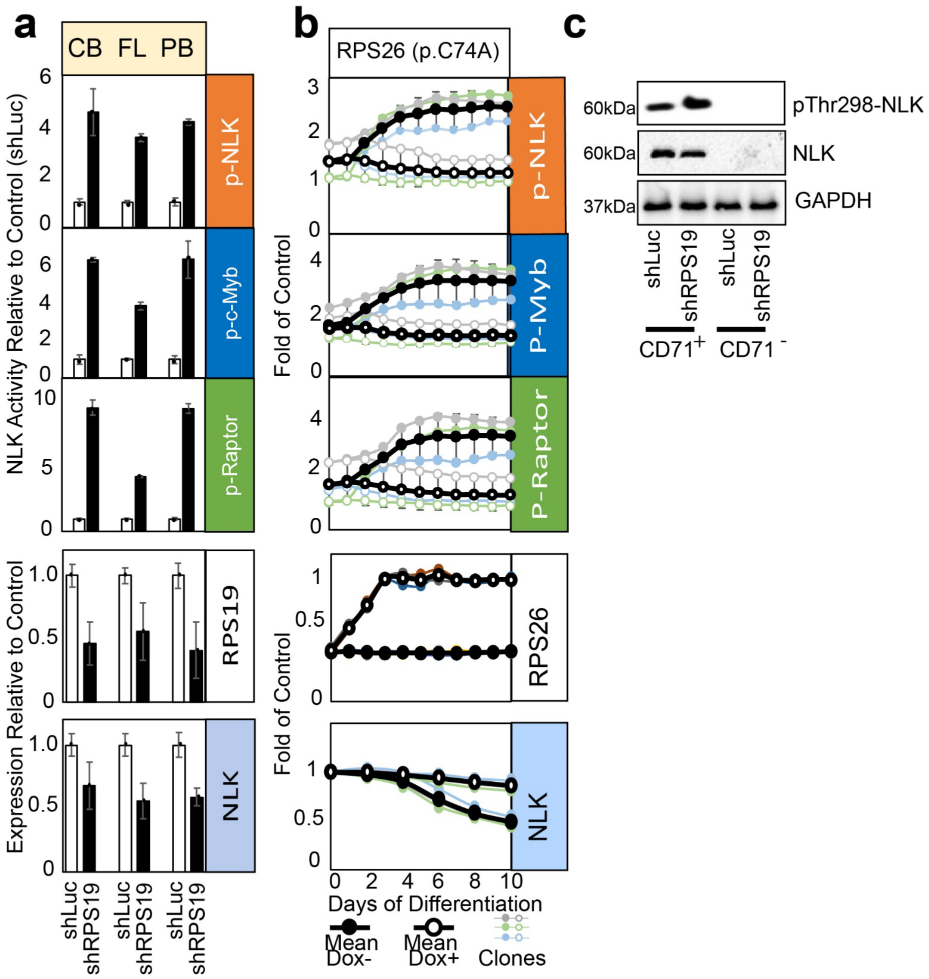

Supplementary Fig. 5. NLK is activated in RPS19-insufficient progenitors from multiple sources and iPS cells from DBA patients. (a) Cord blood (CB), fetal liver (FL) and peripheral blood (PB) CD34+ progenitors were transduced with shRNA against luciferase (white bars) or RPS19 (black bars) and, after sorting, were differentiated for 10 days. Lysates were subjected to NLK kinase assay and the expression of NLK and RPS19 determined by qRT-PCR. (b) iPS cells were generated as described in materials and methods from a DBA patient carrying a mutation in the RPS16 gene (p.C74A) and 3 clones were transduced with tet-on RPS26. After generation of CD34+ HSC-like progenitors, cells were differentiated in the presence (open circles) or absence (closed circles) of doxycycline and assessed for NLK activity and expression, as well as RPS19 expression. Individual clones are indicated in grey, blue and green, and the mean is indicated as a thicker, black line. (c) Intracellular NLK phosphorylation at Thr298 (upper), total NLK (middle) and GAPDH was assessed by Western blot analysis after iPSC-derived HSCs were differentiated in erythroid media for 10 days and sorted into CD71+ and CD71- populations.

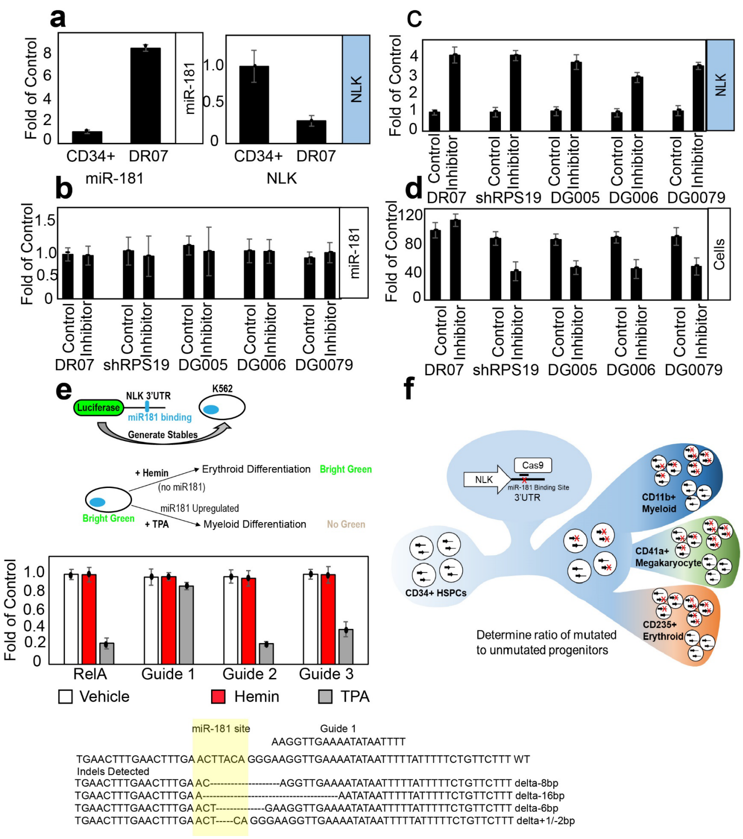

Supplementary Fig. 6. miR-181 expression inversely correlates with NLK expression. (a) CB CD34+ HSPC and control LCL (DR07) were lysed and assessed for miR-181 and NLK expression by qRT-PCR. Control (DR07), control transduced with shRNA against RPS19 (DR07+shRPS19, and LCLs derived from DBA patients (DG005, DG006 and DG0079) were transfected with miR-181 inhibitor and cultured for 4 days prior to lysis and assessment for miR-181 (b) and NLK (c) expression. (d) Prior to lysis cell proliferation was determined by hemacytometer. (e) Diagrammatic representation of luciferase- NLK 3'UTR fusion that was transfected into K562 cells. (Upper) Schematic representation of assay to screen sgRNAs. K562 stable clones electroporated with sgRNAs were differentiated towards erythroid or myeloid lineages, by Hemin or TPA respectively. As miR181 is induced in megakaryocyte and myeloid progenitors, luciferase activity should be diminished in clones with an intact miR181 binding site. Luciferase activity in TPA-treated clones that is equivalent to hemin treatment, indicates a disrupted miR181 binding site. (Middle) K562 cells stably expressing luciferase-NLK 3'UTR were electroporated with sgRNA and Cas9 and allowed to recover for 24 h. Cells were then treated with hemin or TPA for 24 h and luciferase activity was assessed. (Lower) The miR-181 binding site within the NLK 3'UTR, the guide sequence and most abundant indels observed after electroporation are indicated. (f) A schematic of the experimental approach to examine the effects of mutating the predicted miR-181 binding site within the NLK 3'UTR. $CD34^{+}$ progenitors were electroporated with Cas9 and 1 of 2 gRNAs. The first generated indels within the miR181 binding site of the NLK 3'UTR (d181), and the second generated indels within the RelA gene (Rel A). After 24h recovery, cultures were transduced with shLuc or shRPS19 and differentiated for 15 days. The data is included in Fig. 5d and e.

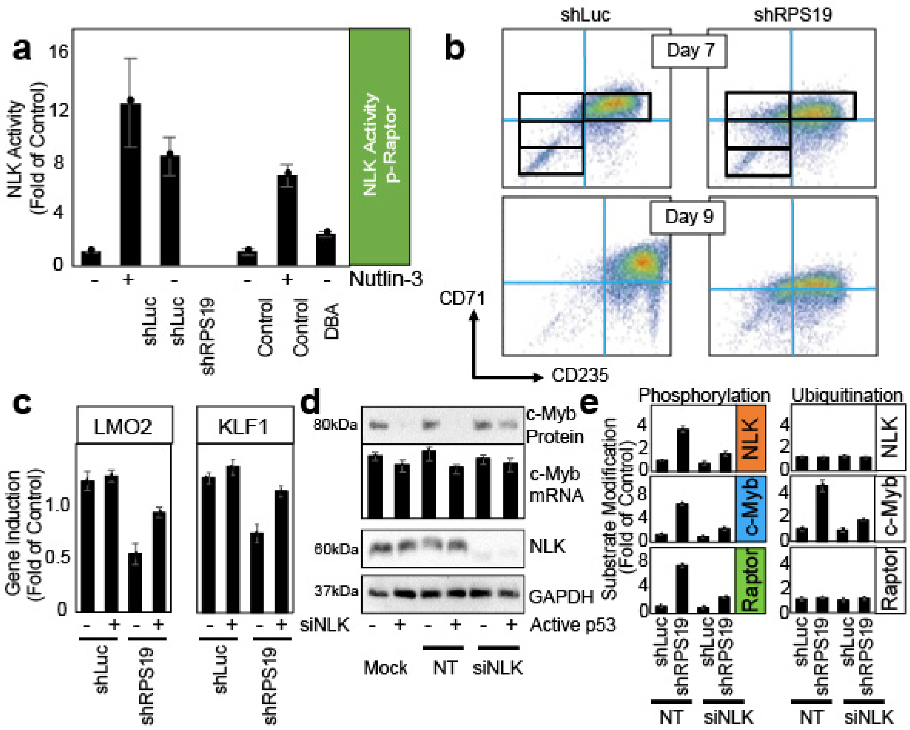

Supplementary Fig. 7. Active NLK phosphorylates c-Myb, leading to ubiquitination, degradation and reduction in LMO2 and KLF1 induction. (a) CD34+ cord blood HSPCs were transduced with shRNA against control (shLuc) or RPS19, along with YFP- and CFP-tagged NLK and allowed to differentiate for 3 days, in the presence or absence of Nutlin-3. In parallel, bone marrow mononuclear cells from healthy donors or DBA patients were transduced with YFP- and CFP-NLK and incubated alone, or with Nutlin-3 for 24 hours. Cells were lysed and NLK in vitro kinase activity was assessed. (b) To compare erythroid differentiation at day 7 and 9 between RPS19-insufficient and control cells we examined CD71 and CD235 staining by flow cytometry. Day 7 samples were gated to include non-erythroid $CD71^{-}CD235^{-}$ populations, MEP- and BFU-E-enriched $CD71^{low}CD235^{-}$ populations, CFU-E-enriched $CD71^{hi}CD235^{-}$ populations and proerythroblast and intermediate erythroblast $CD71^{hi}CD235^{+}$ populations.(c) Cord blood CD34+ progenitors were transduced with shRNA against luciferase or RPS19 and siRNA against NLK or a non-targeting sequence and differentiated in erythroid media for 12 days. Expression of the c-Myb-dependent genes LMO2 (left panel) and KLF1 (right panel) was examined by qRT-PCR and fold of control are shown. (d) Kp53A1 cells alone, or transfected with plasmids expressing NT or siNLK were cultured for 30h at 37 or 32°C prior to lysis and western blotting for c-Myb, NLK and GAPDH. Myb mRNA levels were determined by qRT-PCR. (e) CD34+ progenitors transduced with shRNA against luciferase (shLuc) or RPS19 (shRPS19) in conjunction with siRNA against NLK (siNLK) or a non-targeting sequence (NT) were differentiated for 5 days. Lysates were incubated for 60 min at 37°C in the presence of immobilized NLK, c-Myb and Raptor prior to assessment of phosphorylation and ubiquitination as described.

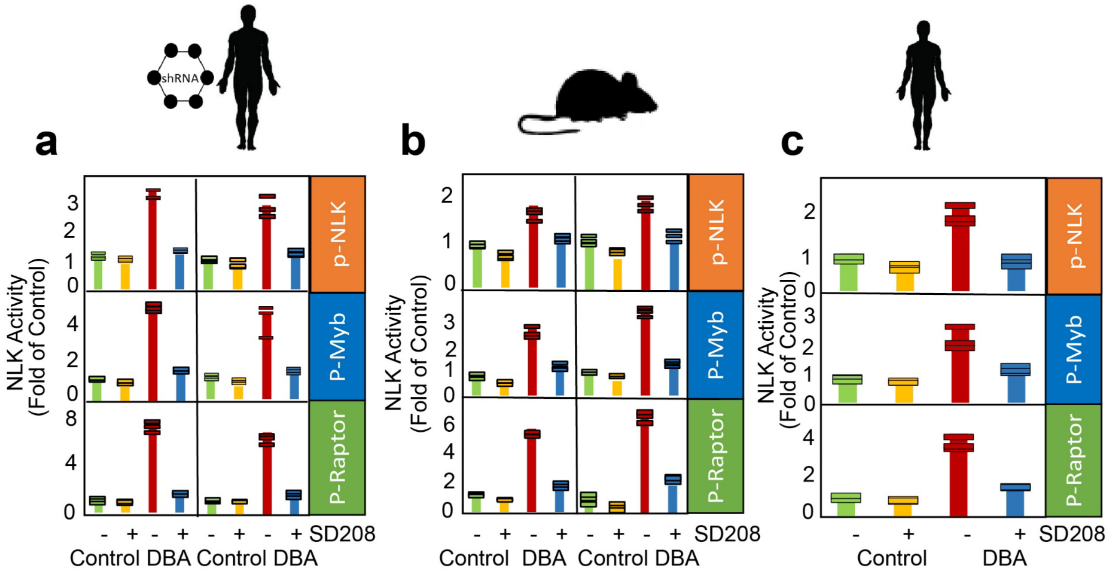

Supplementary Fig. 8. SD208 inhibits NLK *in vitro* kinase activity. (a) Human cord blood CD34$^+$ progenitors were transduced with lentivirus co-expressing GFP with shRNA against luciferase (shLuc), RPS19 (shRPS19) or RPL11 (shRPL11). After 36 hours, GFP$^+$ cells were differentiated in erythroid media in the presence or absence of 5 μM SD208 for 15 days. Approximately 3000 cells were removed from differentiating treatments at day 6 and *in vitro* NLK activity was assessed. Values were normalized to reflect a fold induction relative to untreated controls (b). Lin$^-$Kit$^+$ hematopoietic progenitors were obtained from three mouse embryos expressing tetracycline-inducible shRNA against RPS19 at day E14.5 or three untreated mice, and thee mature RPL11$^{+/+}$ or three mature RPL11$^{+/lox}$ mice treated with tamoxifen for eight weeks. Cells were grown in the presence or absence of doxycycline and/or SD208 for 5 days and NLK *in vitro* activity was assessed. (c) CD34$^+$ HSPCs were isolated from three healthy control and three DBA patient mononuclear bone marrow aspirates by magnetic bead sorting and differentiated in the presence or absence of SD208 for 5 days and *in vitro* kinase activity was assessed as above.

Supplementary Table 1. Primer List

**Gene**	**Sequences**	
h7SL	Fwd – ATCGGGTGTCCGCACTAAGTT	Rvs - CAGCACGGGAGTTTTGACCT
hNLK	Fwd – CAGCCATATTTCCATCACC	Rvs - GACAACACCAAAGGCTCCAT
mNLK	Fwd – CACTCGCATCATCCGCAAC	Rvs - TTCCCGGAAGACTCTTTTGC
hRPS19	Fwd – GCCTGGAGTTACTGTAAAAGACG	Rvs - CCCATAGATCTTGGTCATGGAGC
mRPS19	Fwd – CAGCACGGCACCGTACC	Rvs - GCTGGGTCTGACACCGTTTTC
hRPL11	Fwd – ATGGCGCAAGATCAAGGGG	Rvs – GACTGTGCAGTGAACAGCAAT
mRPL11	Fwd – GCATAATCATTGGTTGGGCCTGA	Rvs - CCGGATGCCAAAGGACCTGAC
hRPS26	Fwd – GGACATTTCTGAAGCGAGCGTC	Rvs - CGATTCCTGACTACTTTGCTGTG
hP53	Fwd – AGGCCTTGGAACTCAAGGAT	Rvs - TGAGTCAGGCCCTTCTGTCT
hmiR-181	Fwd – TTCAACGCTGTCGGTGA	Rvs - GAACATGTCTGCGTATCTC
hc-Myb	Fwd – ATTGCCAATTATCTCCGAATC	Rvs - CCAATTCTCCCCTTTAAGTGCT
hLMO2	Fwd – TACTTCCTGAAAGCCATCGACC	Rvs - GATCCCATTGATCTTGGTCCAC
hKLF1	Fwd – CATCAGCACGGTTGTTGCTGT	Rvs – CACAAACTTCACGTTGGCCTG

Mutagenesis

Kinase Dead NLK – CGCTCACGAAGATGCCCAACGTCTTCCAGAATCTGGTC

NLKesc – TTAGATGAATCACGTCACATGACTCAAGAGGTAGTTACTCAG

NLK 3'UTR miR181 binding site – CTCGAGCGATGCGGCCGGTTTGCAAAGTCAGGATA
