# Supplementary material for: Diamond Blackfan anemia is mediated by hyperactive Nemo-like kinase
Source: Nat Commun. 2020 Jul 3;11:3344. doi: 10.1038/s41467-020-17100-z (PMC7334220; doi:10.1038/s41467-020-17100-z)
Supplement: Supplementary file 2 — Reporting Summary [file 41467_2020_17100_MOESM2_ESM.pdf]

nature research

Corresponding author(s): Kathleen M Sakamoto

Last updated by author(s): Mar 27, 2020

Reporting Summary

Nature Research wishes to improve the reproducibility of the work that we publish. This form provides structure for consistency and transparency in reporting. For further information on Nature Research policies, see Authors & Referees and the Editorial Policy Checklist .

Statistics

For all statistical analyses, confirm that the following items are present in the figure legend, table legend, main text, or Methods section.

n/a	Confirmed	
☐	☒	The exact sample size (*n*) for each experimental group/condition, given as a discrete number and unit of measurement
☐	☒	A statement on whether measurements were taken from distinct samples or whether the same sample was measured repeatedly
☐	☒	The statistical test(s) used AND whether they are one- or two-sided<br>*Only common tests should be described solely by name; describe more complex techniques in the Methods section.*
☒	☐	A description of all covariates tested
☐	☒	A description of any assumptions or corrections, such as tests of normality and adjustment for multiple comparisons
☐	☒	A full description of the statistical parameters including central tendency (e.g. means) or other basic estimates (e.g. regression coefficient) AND variation (e.g. standard deviation) or associated estimates of uncertainty (e.g. confidence intervals)
☐	☒	For null hypothesis testing, the test statistic (e.g. *F*, *t*, *r*) with confidence intervals, effect sizes, degrees of freedom and *P* value noted<br>*Give P values as exact values whenever suitable.*
☒	☐	For Bayesian analysis, information on the choice of priors and Markov chain Monte Carlo settings
☒	☐	For hierarchical and complex designs, identification of the appropriate level for tests and full reporting of outcomes
☒	☐	Estimates of effect sizes (e.g. Cohen's *d*, Pearson's *r*), indicating how they were calculated

Our web collection on statistics for biologists contains articles on many of the points above.

Software and code

Policy information about availability of computer code

Data collection	no software was used
Data analysis	Flow cytometry data was analyzed by using FlowJo Software, v.9.7.2.

For manuscripts utilizing custom algorithms or software that are central to the research but not yet described in published literature, software must be made available to editors/reviewers. We strongly encourage code deposition in a community repository (e.g. GitHub). See the Nature Research guidelines for submitting code & software for further information.

Data

Policy information about availability of data

All manuscripts must include a data availability statement. This statement should provide the following information, where applicable:

- Accession codes, unique identifiers, or web links for publicly available datasets
- A list of figures that have associated raw data
- A description of any restrictions on data availability

The authors declare that [the/all other] data supporting the findings of this study are available within the paper (and its supplementary information files). Additional information and further discussion are available from the corresponding author upon reasonable request.

Field-specific reporting

Please select the one below that is the best fit for your research. If you are not sure, read the appropriate sections before making your selection.

☒ Life sciences ☐ Behavioural & social sciences ☐ Ecological, evolutionary & environmental sciences

Life sciences study design

All studies must disclose on these points even when the disclosure is negative.

Sample size	Experiments were repeated independently a minimum of three times. Within each independent repeat, conditions and/or treatments were performed in duplicate or triplicate.
Data exclusions	All experiments in which internal controls performed as appropriate are included. Experimental results were only excluded if internal controls failed, or experiments were terminated prematurely due to contamination of experimental error.
Replication	In the case of significant deviation between repeated experiments, further repeats were performed. If a variable could be attributed to the deviation, data include represents that in which the variable was controlled for. If no variable could be determined to account for the variability, all data was included with an increased number of repeats.
Randomization	Each repeat was performed independently a minimum of 3 times. When possible, multiple students, postdoctoral fellows and technicians performed various elements, and all data was discussed collectively in an attempt to maximize consistency and integrate data from multiple groups across the United States, Spain and Sweden.
Blinding	n cases when some ambiguity can be introduced (eg. colony assays), multiple postdoctoral fellows with extensive experience were consulted to blindly score experiments to eliminate bias. Numerous experiments were also performed/repeated by students with limited knowledge of anticipated results.

Reporting for specific materials, systems and methods

We require information from authors about some types of materials, experimental systems and methods used in many studies. Here, indicate whether each material, system or method listed is relevant to your study. If you are not sure if a list item applies to your research, read the appropriate section before selecting a response.

Materials & experimental systems

n/a	Involved in the study	
☐	☒	Antibodies
☐	☒	Eukaryotic cell lines
☒	☐	Palaeontology
☐	☒	Animals and other organisms
☐	☒	Human research participants
☒	☐	Clinical data

Methods

n/a	Involved in the study	
☒	☐	ChIP-seq
☐	☒	Flow cytometry
☒	☐	MRI-based neuroimaging

Antibodies

Antibodies used	Western blotting and immunoprecipitation antibodies against RPS19 (#AB40833; Abcam; lot:518525), NLK (#AB97642; Abcam; lot:GR172200-31) c-Myb (#12319; Cell Signaling; (D2R4Y), raptor (#AB26264; Abcam lot:GR20363-5), phosphor-Serine (#525280; Calbiochem; lot: B32644), S6K (#9202; Cell Signaling; lot: 7), and 4E-BP1 (#9452; Cell Signaling; lot:10), and GAPDH (#MAB374; Millipore; lot: 3090497) were used according to manufacturer's instructions. Antibodies for flow cytometry: CD235 AP (Cat: 306607, Biolegend; lot: B242428, clone: H1R2), CD11b PE/Cy5 (cat: 101209 Biolegend; lot: B225288, clone: M1/70), CD41a FITC (Cat: 303703, Biolegend, Lot: B208418 clone: H1P8) Ter119 PE (cat:553673 Pharmingen lot:0000055444, clone: Ly-7b).
Validation	All antibodies were validated by western blot. Samples were run against a size ladder and bands were compared with predicted size and the pattern of bands compared with manufacturer supplied information. In the case of NLK, RPS19 and RPL11, antibody specificity was further assessed after gene silencing.

Eukaryotic cell lines

Policy information about cell lines

Cell line source(s)	Kp53A1 cells were obtained from Javier Leon. K562 were purchased from ATCC. iPSCs and iPSC-derived HSCs in collaboration with Dr Hiromitsu Nakauchi, as per their published techniques.
Authentication	HSPCs were obtained from listed sources and were purified by CD34 status by magnetic separation. Kp53A1 were validated by western blot analysis of p53, p21, c-Myc and c-Myb status at 32 and 37 degrees celsius.
Mycoplasma contamination	Cell lines were routinely examined for mycoplasma contamination anually. No contamination was detected.
Commonly misidentified lines (See ICLAC register)	No cell lines listed on the ICLAC register were utilized in this study

Animals and other organisms

Policy information about studies involving animals; ARRIVE guidelines recommended for reporting animal research

Laboratory animals	The RPS19-deficient mouse model contains a doxycycline-regulatable Rps19-targeting shRNA (shRNA-D) located downstream of the collagen A1 locus, allowing dose-dependent downregulation of Rps19 expression (PMID: 21435508). Inducible RPL11 heterozygous deletion mice36 were fed a standard chow diet ad libitum. When indicated, standard chow diet was replaced by tamoxifen diet (Teklad, Harlan Laboratories) to induce activation of the CreERT2 transgene.
Wild animals	No wild animals were utilized in this study
Field-collected samples	No field-collected animals were utilized in this study
Ethics oversight	Mice were maintained at the Lund University animal facility (Sweden) and all animal experiments were performed with consent from the Lund University animal ethics committee or were maintained at the Spanish National Cancer Research Centre (CNIO) under specific pathogen-free conditions, in agreement with the recommendations of the Federation of European Laboratory Animal Science Association (FELASA). All animal procedures were evaluated and approved by the Ethical Committee of the Carlos III Health Institute, Madrid, Spain (#54-2013-v2).

Note that full information on the approval of the study protocol must also be provided in the manuscript.

Human research participants

Policy information about studies involving human research participants

Population characteristics	3 unrelated DBA probands with the following mutations in RPS19: c.3G>A, p.Met1Lys, c.105_106ins A, p.Thr36Asnfs*16 and c.185 G>A, pArg62Gln were obtained from Boston Childern's Hospital. A patient diagnosed with DBA with unknown genetic background and control were obtained from UCLA.
Recruitment	Bone Marrow Aspirates from control and DBA patients were obtained under IRB approval.
Ethics oversight	IRB approval from UCLA (IRB:11062) and Boston Children's Hospital (IRB: 06-08-0384)

Note that full information on the approval of the study protocol must also be provided in the manuscript.

Flow Cytometry

Plots

Confirm that:

☒ The axis labels state the marker and fluorochrome used (e.g. CD4-FITC).

☒ The axis scales are clearly visible. Include numbers along axes only for bottom left plot of group (a 'group' is an analysis of identical markers).

☒ All plots are contour plots with outliers or pseudocolor plots.

☒ A numerical value for number of cells or percentage (with statistics) is provided.

Methodology

Sample preparation	For cell surface flow cytometry, cells were incubated with human Fc receptor binding inhibitor (#14-9161-73; eBioscience, Inc.) followed by primary antibodies.
Instrument	Data were collected on a DxP10 flow cytometer (Cytek).
Software	Data was analyzed by using FlowJo Software, v.9.7.2.
Cell population abundance	Human cells. After differentiation, populations were counted and 10,000 - 20,000 were analyzed for extracellular staining of hematopoietic markers. The proportion was multiplied by the total cell count to derive the abundance. Purity was assessed by staining for CD235, CD41a and CD11b.
Gating strategy	Using the FSC/SSC gating, debris was removed by gating on the main cell population. Positivity threshold for each cell line was defined on the basis of sample with no antibody present. Identical positivity threshold was applied to all samples within cell line. A representative plot and gating is provided with Figure 5C and Figure S7D as well as Fgure S13a

☒ Tick this box to confirm that a figure exemplifying the gating strategy is provided in the Supplementary Information.
